# Bamboo (Poales, Poaceae): An Important Maintainer of Immature Mosquitoes (Diptera: Culicidae) in a Biodiversity Hotspot in the City of Rio de Janeiro, Brazil

**DOI:** 10.3390/life14030351

**Published:** 2024-03-07

**Authors:** Manuella Pereira Cerqueira Leite, Rayane Dias, Paulo José Leite, Shayenne Olsson Freitas Silva, Hélcio Reinaldo Gil-Santana, Roger Pimentel Barbosa, Cecilia Ferreira de Mello, Jeronimo Alencar

**Affiliations:** 1 Laboratório de Diptera, Instituto Oswaldo Cruz (Fiocruz), Avenida Brasil 4365, Manguinhos, Rio de Janeiro 21040-360, Brazil; manuellapcleite@gmail.com (M.P.C.L.); rayanefdias@edu.unirio.br (R.D.); paulo.leite@ioc.fiocruz.br (P.J.L.); shayenneolsson@gmail.com (S.O.F.S.); helciogil@uol.com.br (H.R.G.-S.); rogerpwolf@gmail.com (R.P.B.); ceciliafmello@gmail.com (C.F.d.M.); 2 Programa de Pós-Graduação em Biologia Animal, Instituto de Biologia, Universidade Federal Rural do Rio de Janeiro, Rio de Janeiro 23890-000, Brazil; 3 Programa de Pós-Graduação em Medicina Tropical, Instituto Oswaldo Cruz (Fiocruz), Avenida Brasil 4365, Manguinhos, Rio de Janeiro 21040-360, Brazil

**Keywords:** Culicidae, immatures, biodiversity, conservation unit, abiotic factors

## Abstract

Although tropical forests are home to most of the global diversity, they suffer from the most significant knowledge gaps concerning their fauna. Despite its high biodiversity, Brazil is facing an alarming destruction of habitats, with species becoming extinct before they can be discovered or described via science. Therefore, there is an urgent need to expand wildlife inventories, including entomofauna surveys. The present study aimed to analyze the bionomic aspects and the influence of abiotic factors on mosquito fauna whose immature phases develop in two bamboo species, *Guadua tagoara* and *Bambusa vulgaris*, in Tijuca National Park, Rio de Janeiro, Brazil. Immatures were collected in 10 artificially drilled bamboo plants, in five stalk internodes per plant, at two sampling points, from March 2022 to March 2023, during 23 collections. A total of 1845 immatures were obtained, 72.14% at sampling point 1 and 27.86% at sampling point 2. Of this, 1162 individuals reached adulthood, belonging to the following species: *Culex iridescens*, *Culex neglectus*, *Haemagogus leucocelaenus*, *Orthopodomyia albicosta*, *Sabethes identicus*, *Sabethes melanonymphe*, *Sabethes purpureus*, *Toxorhynchites bambusicola*, *Toxorhynchites* sp., *Trichoprosopon compressum*, *Trichoprosopon pallidiventer*, *Wyeomyia arthrostigma*, *Wyeomyia codiocampa*, *Wyeomyia lutzi*, *Wyeomyia oblita*, *Wyeomyia personata*, *Wyeomyia serrata*, and *Wyeomyia* sp. The Tijuca National Park is a tourist spot and receives a large number of visitors. Thus, humans can become an accessible food source for mosquitoes in this area, making the species survey critical since important arbovirus vectors have been recorded in Rio de Janeiro.

## 1. Introduction

Mosquitoes of the family Culicidae are of significant medical and veterinary importance given their hematophagous habit, wide and persistent distribution, and the various diseases they transmit, such as dengue, chikungunya, yellow fever, and malaria, which are serious dangers to Public Health [1].

Culicidae larval habitats consist of bodies of water that differ in size, quantity, and physicochemical composition. The immature forms, therefore, occupy various types of habitats. The development of the immature forms of dendrotelmata sylvatic mosquitoes is restricted to certain types of natural habitats formed by the internodes in bamboo (Poales: Poaceae). This plant, which provides them with a small habitat, is commonly found in the tropical and subtropical regions of the world [2]. Several native or exotic bamboo species, mainly of the genus *Bambusa* Schreb. have been found in forest fragments of the Atlantic Forest in Brazil [3]. Some mosquito species are highly flexible in adapting to larval habitats and are found in various habitats, whereas others are more restrictive when choosing sites for immature development [4].

When drilled naturally, bamboo can serve as a microhabitat for wildlife, thus allowing studies on ecological succession [5]. Rainfall carries organic particles through the internode of the plants to their artificial or natural orifices, promoting the development of microorganisms and becoming a source of nutrients for the larval stage of the Culicidae.

Although there is some information in the scientific literature regarding culicids that use bamboo internodes as larval habitats [6,7,8], research in this area is still scarce. It is therefore critical to investigate the preferences of Culicidae that use this type of larval habitat.

Müller et al. [9,10] discussed the bionomy of mosquitoes that use bamboo internodes as habitats for immature forms, noting that these biotopes are important in the maintenance of the culicid fauna, and they considered the presence of important species from an epidemiological point of view.

The availability of food resources, the presence of microorganisms, and the pH, temperature, salinity, composition and content of organic matter, and intra- and interspecific interactions influence the development and survival of immature stages [11,12].

The present study aims to provide information about the bionomy of mosquitoes that select bamboo as larval habitats. For this purpose, we evaluated the diversity, abundance, sexual proportion, and influence of the abiotic factors temperature, humidity, and rainfall on the composition of mosquito fauna whose immatures are raised in artificially drilled bamboo internodes at the Tijuca National Park (TNP), city of Rio de Janeiro, Brazil.

## 2. Materials and Methods

### 2.1. Ethics Statement

The permanent license for the collection, capture, and transport of zoological material was granted by the Chico Mendes Institute for Biodiversity Conservation (Chico Mendes de Conservação da Biodiversidade—ICMBio) and the Biodiversity Information Permission Syculm (Sistema de Permissão de Informações sobre Biodiversidade—SISBIO) under No. 81388-3. All members of the collection team were properly vaccinated against yellow fever. 

### 2.2. Study Area 

The TNP is a federal conservation unit in Rio de Janeiro, representing one of the remaining small fragments in the Atlantic Forest. As its name implies, the park is property of the Federal Government and is classified as a Full Protection Conservation Unit. As a National Park, various activities such as scientific research and public visitation are allowed.

According to Machado (2003) [13], the physiographic landscape of the TNP contains perennial humid sub-mountain tropical forest, representing an ecosystem that is in the process of degradation and erosion acceleration. 

The climate of the Tijuca Massif is classified as subhumid temperate due to its geographical orientation, with abundant rainfall. The average annual rainfall is approximately 2500 mm, and the dry season runs from May to August [14].

The park contains approximately 1619 plant species and over 320 vertebrate animal species, and it plays a critical role in species conservation. Its forest covers an area of approximately 3950 hectares. The vegetation that covers the Tijuca Massif interacts directly with the urban space, making the park an important tourist spot in the city, with over 3 million visits per year. 

### 2.3. Experimental Design

**Figure 1:**
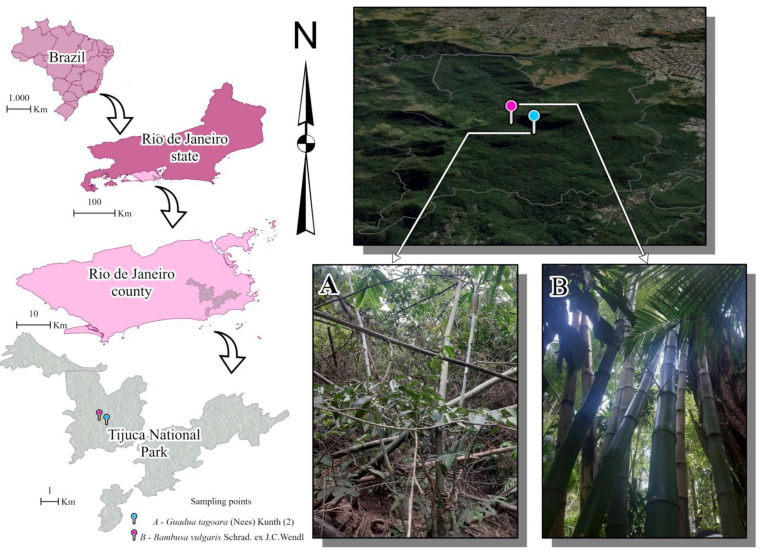
Sampling site in the Tijuca National Park, city of Rio de Janeiro, Brazil. Sampling points: (**A**) *Guadua tagoara* (Nees) Kunth; (**B**) *Bambusa vulgaris* Schrad. ex J.C.Wendl.

The internodes of the bamboo specimens were drilled in February and between March 2022 and March 2023. Immatures were collected at two sampling points, from 10:00 a.m. to 1:00 p.m., with a sampling effort of 23 collections. The sample area contained two sample sites, named sampling point 1 (Figure 1A) and sampling point 2 (Figure 1B), with a standardized sampling time of 90 min at each of them, totaling 180 min per sample and a total sampling effort of 69 h (Figure 1).

Sampling point 1 (22°56′52.1″ S and 43°17′29.3″ W) is located approximately 30 m from the edge of Bom Retiro square, which contains tree species, undergrowth, and a large number of herbaceous plants and the bamboo *Guadua tagoara* (Nees) Kunth bamboo, which was analyzed in this study. 

Sampling point 2 (22°57′00.5″ S and 43°17′15.7″ W) is located at the edge of the forest, containing vegetation that is in an advanced stage of regeneration as well as exotic species, in addition to medium-sized shrub and herbaceous tree species. The selected bamboo plants belong to *Bambusa vulgaris* Schrad. ex J.C.Wendl. and are located about 10 m downhill from Major Archer Road. 

The bamboo specimens were drilled with holes measuring five millimeters in diameter to serve as a microhabitat for the mosquito fauna. Bamboo is cylindrical and divided into sections. The section between two nodes is called the internode. The internodes are hollow in most bamboo species, such as *B. vulgaris* and *G. tagoara*. A perforation was placed in each internode, 25 cm from the lower node of the plant. Only the internodes of live internodes were used, drilled circularly with a rechargeable electric drill. When choosing the drilling pattern, we considered the behavior of insects able to use internodes and the drilling patterns required for their entry and exit.

At each sampling point, five internodes of five bamboo plants were drilled (a total of 25 larval habitats per collection point). Every 15 days, the water from the internodes was removed through manual siphoning to obtain the immature specimens. The physical–chemical characteristics of the water from the bamboo internodes (pH and temperature) were evaluated. To measure the abiotic variables, a portable measuring device from the Akso brand, model AK90 (São Leopoldo, Brazil) was used. The pH and temperature were recorded at the same time. The water was poured into polyethylene trays, and the larvae and pupae were quantified and removed with a pipette so that they could be packed separately in vials and transported to a laboratory for breeding until becoming adults, following the methodology of Müller et al. (2009) [15]. The contents of each sample were transferred to small vials, and the larvae were kept alive with the water of the internodes and periodically supplemented with dechlorinated water in a controlled experimental environment at a temperature of 28 °C and air humidity between 75 and 90%. The specimens were monitored daily until the immatures reached adulthood. Species were identified using the direct observation of the morphological characteristics of the adults under an optical microscope and the consultation of the respective descriptions/diagnoses of the species, using dichotomous keys and information from the works of Lane (1953) [16,17], Consoli and Lourenço-de-Oliveira (1994) [18], and Forattini (2002) [19]. Abbreviations of genus and subgenus names follow the proposal by Reinert (2001) [20]. After species identification, all specimens were incorporated into the Entomological Collection of the Oswaldo Cruz Institute, Fiocruz, under the title “Parque Nacional da Tijuca”.

### 2.4. Statistical Analyses

The following ecological indices were used to evaluate, analyze, and compare the diversity of mosquitoes at each sampling point: diversity, dominance, richness, and equability. The Shannon Diversity Index was used to evaluate immature abundance collected at each sampling point, which comprised different bamboo species: *Guadua tagoara* (sampling point 1) and *Bambusa vulgaris* (sampling point 2). The Simpson Dominance Index was used to measure the probability of two individuals randomly selected in the sample belonging to the same species; higher values of the latter index imply lower diversity levels. The richness index was used to describe the total number of different mosquito species found at each sampling point. Equability, also known as evenness, reflects the uniformity of abundance among different mosquito species within each sampling point. The linear regression test was used to analyze the correlations between immatures and the following variables: temperature, pH, and water volume variables. The differences between the abundance of males and females were evaluated with the t-test. All analyses were conducted using software PAST version 4.05.

## 3. Results 

During the sampling period, 1845 immature culicids were collected, 1331 (72.14%) at sampling point 1 and 514 (27.86%) at sampling point 2. Of this total, 1162 reached adulthood, being distributed as follows: *Culex iridescens* Lutz (*n* = 119; 10.2%); *Culex neglectus* Lutz (*n* = 394; 33.9%); *Haemagogus leucocelaenus* (Dyar & Shannon) (*n* = 4; 0.3%); *Orthopodomyia albicosta* (Lutz) (*n* = 257; 22.1%); *Sabethes identicus* Dyar & Knab (*n* = 164; 14.1%); *Sabethes melanonymphe* Dyar (*n* = 2; 0.2%); *Sabethes purpureus* (Theobald) (*n* = 3; 0.3%); *Toxorhynchites bambusicola* (Lutz & Neiva) (*n* = 1; 0.1%); *Toxorhynchites* sp. Theobald (*n* = 1; 0.1%); *Trichoprosopon compressum* Lutz, 1905 (*n* = 1; 0.1%); *Trichoprosopon pallidiventer* (Lutz) (*n* = 7; 0.6%); *Wyeomyia arthrostigma* (Lutz) (*n* = 10; 0.9%); *Wyeomyia codiocampa* Dyar & Knab (*n* = 1; 0.1%); *Wyeomyia lutzi* (Costa Lima) (*n* = 138; 11.9%); *Wyeomyia oblita* (Lutz) (*n* = 48; 4.1%); *Wyeomyia personata* (Lutz in Bourroul) (*n* = 2; 0.2%); *Wyeomyia serrata* (Lutz) (*n* = 1; 0.1%); *Wyeomyia* sp. Theobald (*n* = 9; 0.8%).

**Figure 2:**
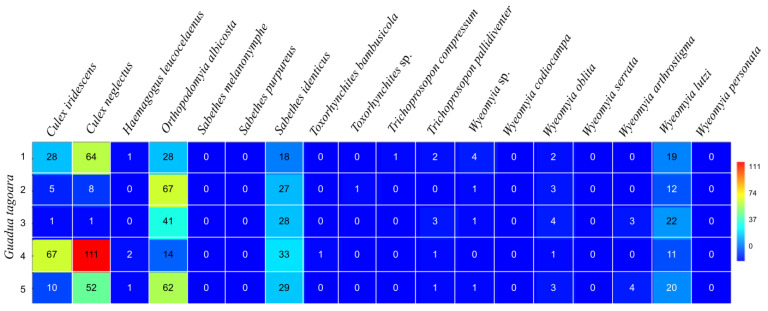
Abundance of immature mosquitoes in *Guadua tagoara* at sampling point 1 (bamboo specimens 1–5) at the Tijuca National Park, city of Rio de Janeiro, Brazil, from March 2022 to March 2023.

**Figure 3:**
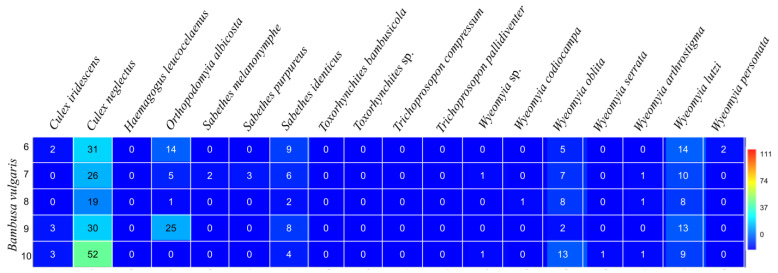
Abundance of immature mosquitoes in *Bambusa vulgaris* at sampling point 2 (bamboo specimens 6–10) at the Tijuca National Park, city of Rio de Janeiro, Brazil, from March 2022 to March 2023.

The highest number of Culicidae specimens was found in sampling point 1 (*G. tagoara*; bamboo specimens 1–5), with 29% of them located on bamboo 4 (*n* = 241), followed by bamboo specimens 5 (*n* = 183; 22%) and 1 (*n* = 167; 20%) (Figure 2). The highest diversity and equitability indices were found in bamboo specimens 1 (H = 1.71; J = 0.74) and 5 (H = 1.65; J = 0.72). For *B. vulgaris* specimens from sampling point 2 (bamboos specimens 6–10), the highest abundance was observed in bamboo 9 (*n* = 81; 24%), followed by bamboo 6 (*n* = 77; 22%) (Figure 3). The highest dominance indices were observed in bamboo specimens 2 (D = 0.36) and 4 (D = 0.31), with a dominance of *Or. albicosta* (bamboo 2) and *Cx. neglectus* (bamboo 4) (Table 1). The highest diversity indices were observed in bamboo 7 (H = 1.74) and 6 (H = 1.60). The highest equitability values were detected in bamboo specimens 6 and 9 (J = 0.82 for both). The highest richness values were observed in bamboo specimens 7 (S = 9) and 10 (S = 8). The highest dominance indices were found in bamboo 10 (D = 0.42) and 8 (0.31), with the dominance of *Cx. neglectus* in both (Table 1). *Culex neglectus*, *Sa. identicus*, *Wy. oblita*, and *Wy. lutzi* were present in all bamboo specimens analyzed.

**Figure 4:**
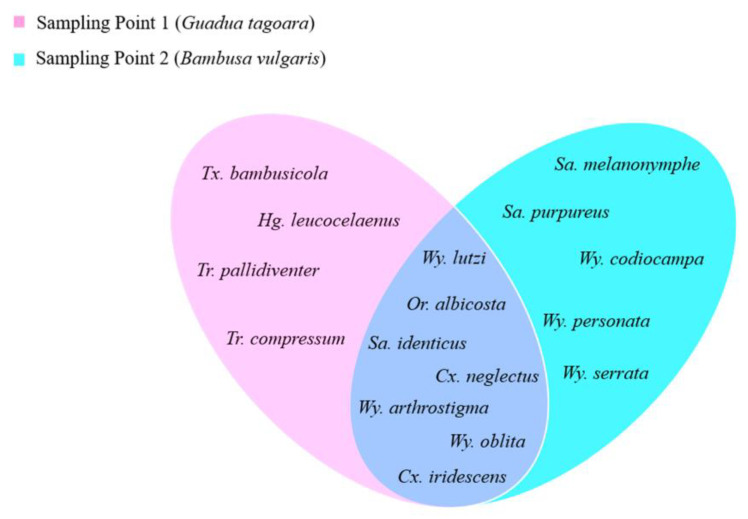
Species found at the two sampling points of the Tijuca National Park, city of Rio de Janeiro, Brazil, from March 2022 to March 2023. *Culex iridescens*, *Culex neglectus*, *Haemagogus leucocelaenus*, *Orthopodomyia albicosta*, *Sabethes melanonymphe*, *Sabethes purpureus*, *Sabethes identicus*, *Toxorhynchites bambusicola*, *Trichoprosopon compressum*, *Trichoprosopon pallidiventer*, *Wyeomyia codiocampa*, *Wyeomyia oblita*, *Wyeomyia serrata*, *Wyeomyia arthrostigma*, *Wyeomyia lutzi*, *Wyeomyia personata*.

Regarding the mosquito species found in the two bamboo species, there was a statistically significant difference between *G. tagoara* and *B. vulgaris* (*p* = 0.006). *Guadua tagoara* had the highest diversity (H = 1.71) and equability indices (J = 0.71). In contrast, *B. vulgaris* had a diversity index of H = 1.63 and an equability index of J = 0.65. *G. tagoara* had 11 species and *B. vulgaris* had 12 species (Figure 4). The dominance index of *B. vulgaris* was higher (D = 0.28), with a greater abundance of *Cx. neglectus*.

The highest abundances of immature culicids were found at sampling point 1, in internodes 2 (*n* = 213; 26%) and 5 (*n* = 201; 25%). The highest diversity indices were observed in internodes 4 (H = 1.79), 1 (H = 1.72), and 2 (H = 1.70), whereas the highest equability indices were detected in internodes 1 (J = 0.88) and 3 (J = 0.84) (Table 2). The highest richness values were found in internodes 2 (s = 11) and 4 (S = 11), and the highest dominance index was observed in internode 5 (D = 0.24), represented by *Cx. neglectus*. 

At sampling point 2, internode 2 showed the highest abundance (*n* = 97; 28%), diversity index (H = 1.76), and richness (S = 10), and the second-highest equability index (J = 0.77). It was followed by internode 1, which also showed a high abundance (*n* = 85; 25%), diversity index (H = 1.73), and richness (S = 09), and the highest equability index (J = 0.79). Internode 5 had the highest dominance index (D = 0.50) (Table 3), also due to *Cx. neglectus*.

**Figure 5:**
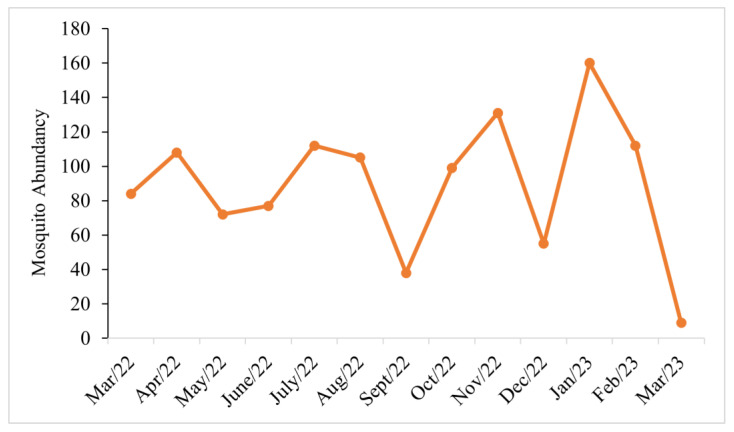
Monthly frequency of immature mosquitoes at the Tijuca National Park, city of Rio de Janeiro, Brazil, from March 2022 to March 2023.

The peaks of immature culicid abundance were found in January 2023 (*n* = 160; 14%) and November 2022 (*n* = 131; 11%). In contrast, in September and December 2022, we detected a decline in the mosquito population. From August to September, there was a 6% decrease in the abundance of collected culicids. In October 2022, the population increased again continuously (5%) until November 2022, declining in December 2022 (*n* = 55), while in the following month (January 2023), it showed its highest peak (*n* = 160), decreasing again in February (*n* = 112) and with the lowest abundance recorded in March 2023 (*n* = 9) (Figure 5).

The highest richness values were observed in July 2022 (S = 11), April 2022 (S = 10), August 2022 (S = 8), and January 2023 (S = 8). The highest diversity indices were found in May, June, and July 2022 (H = 1.7 for the three of them), August 2022 (H = 1.6), and April 2022 (H = 1.5). Regarding the equability index, the highest values were found in June 2022 (J = 0.9) and May 2022, August 2022, and October 2022 (J = 0.8 for all three). On the other hand, the highest rates of dominance were found in March 2022 (D = 0.7), driven by the presence of *Sa. identicus* (*n* = 70) (Table 4).

**Figure 6:**
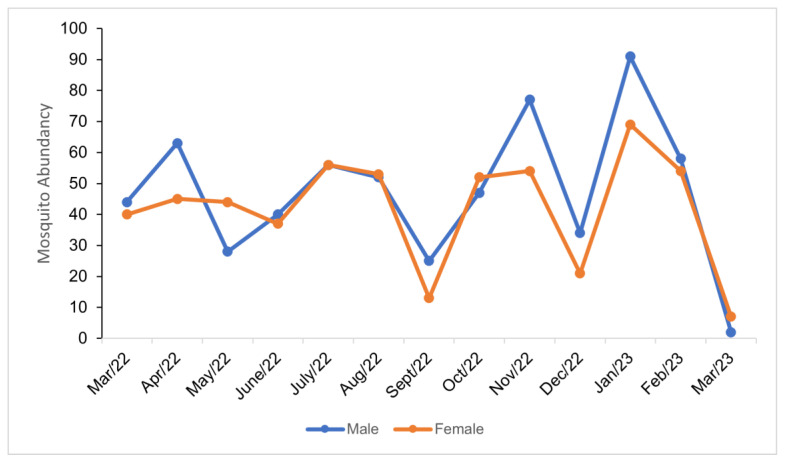
Monthly abundance of male and female mosquitoes at the Tijuca National Park, city of Rio de Janeiro, Brazil, between March 2022 and March 2023.

During the sampling period, the number of males found was 6% higher than that of females, although this was not statistically significant. The months with the highest number of males were March, April, June, September, November, and December 2022, and January and February 2023, while the highest number of females was found in May, August, and October 2022, and March 2023 (Figure 6). 

**Figure 7:**
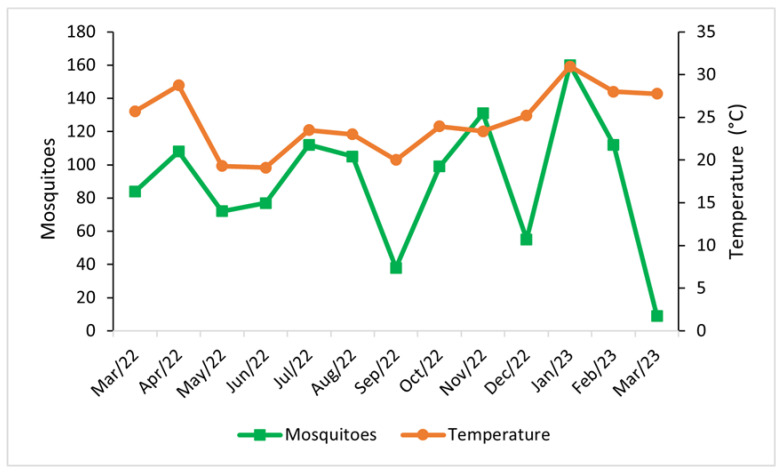
Monthly abundance of mosquitoes and temperature at the Tijuca National Park, city of Rio de Janeiro, Brazil, between March 2022 and March 2023.

The largest percentage difference in males and females occurred in September 2022 (32%), December 2022 (24%), November 2022 (18%), and April 2022 (17%), with males predominating during each of those months. Conversely, a greater number of females was found in March 2023 (56%) and May 2022 (22%). Mosquito abundance followed the temperature pattern with higher peaks at higher temperatures and decreasing with lower temperature values (Figure 7). 

**Figure 8:**
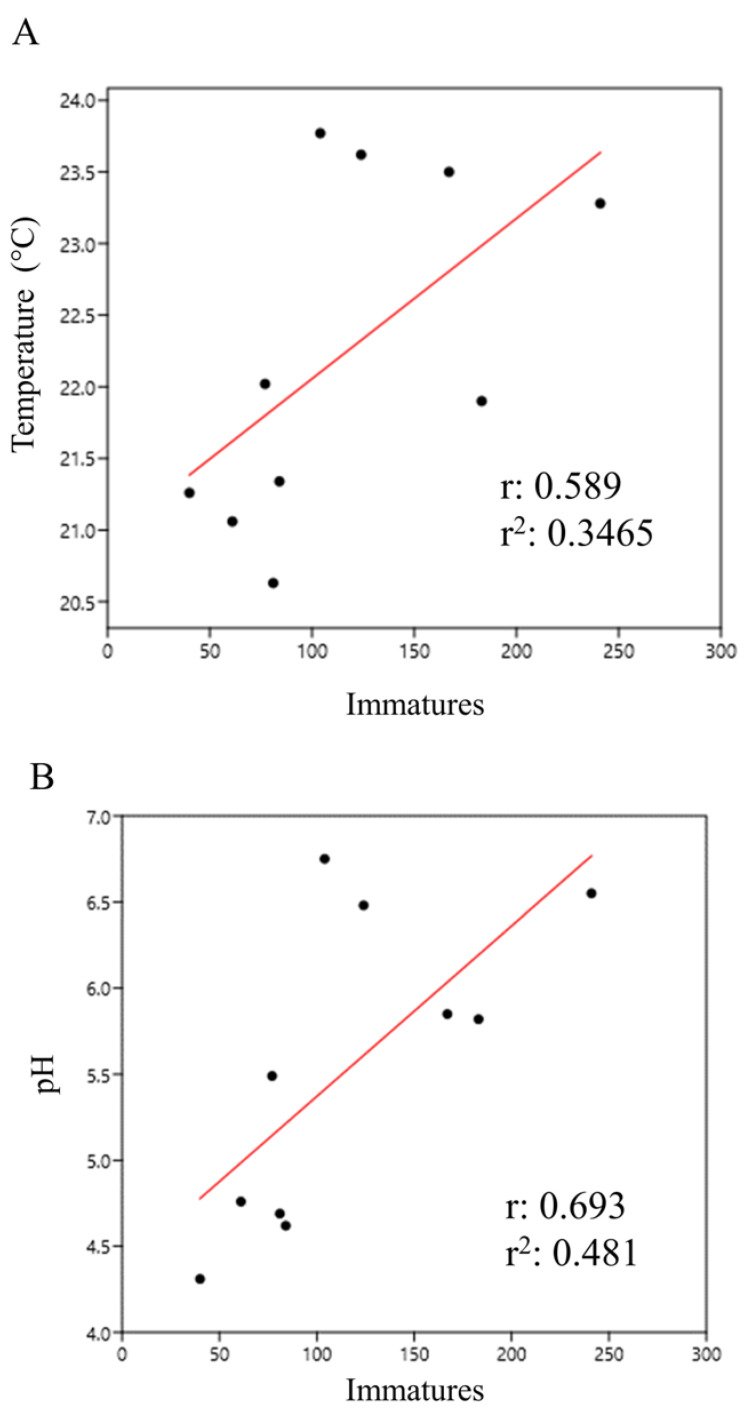
Abundance of immatures in bamboo plants correlated with temperature (**A**) and pH (**B**) at the Tijuca National Park, city of Rio de Janeiro, Brazil, from March 2022 to March 2023.

In general, immature abundance in bamboo plants showed a moderate positive correlation with temperature (r = 0.59) and pH (r = 0.694) (Figure 8A,B).

**Figure 9:**
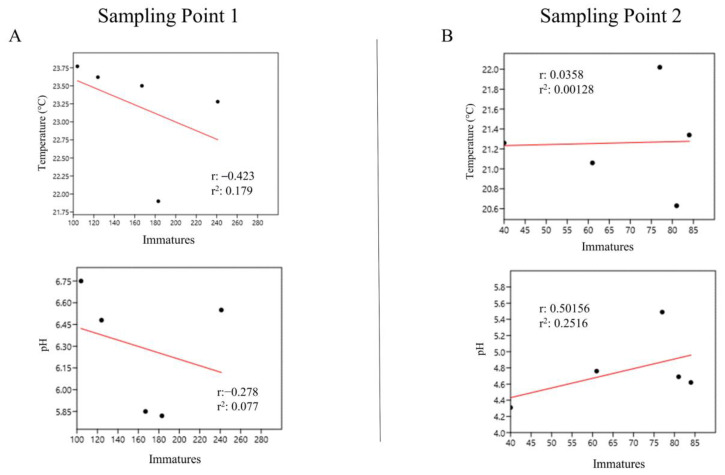
Abundance of immatures in bamboo plants at sampling points 1 (**A**) and 2 (**B**) correlated with temperature and pH at the Tijuca National Park, city of Rio de Janeiro, Brazil, from March 2022 to March 2023.

At sampling point 1 (bamboos 1 to 5, from the species *G. tagoara*), we found a weak and negative correlation between immature abundance and temperature (r = −0.423) and pH (r = −0.278) (Figure 9A). At sampling point 2 (bamboos 6 to 10, from the species *B. vulgaris*), there was a weak and positive correlation between immature abundance and temperature (r = 0.036) and pH (r = 0.502) (Figure 9B).

**Figure 10:**
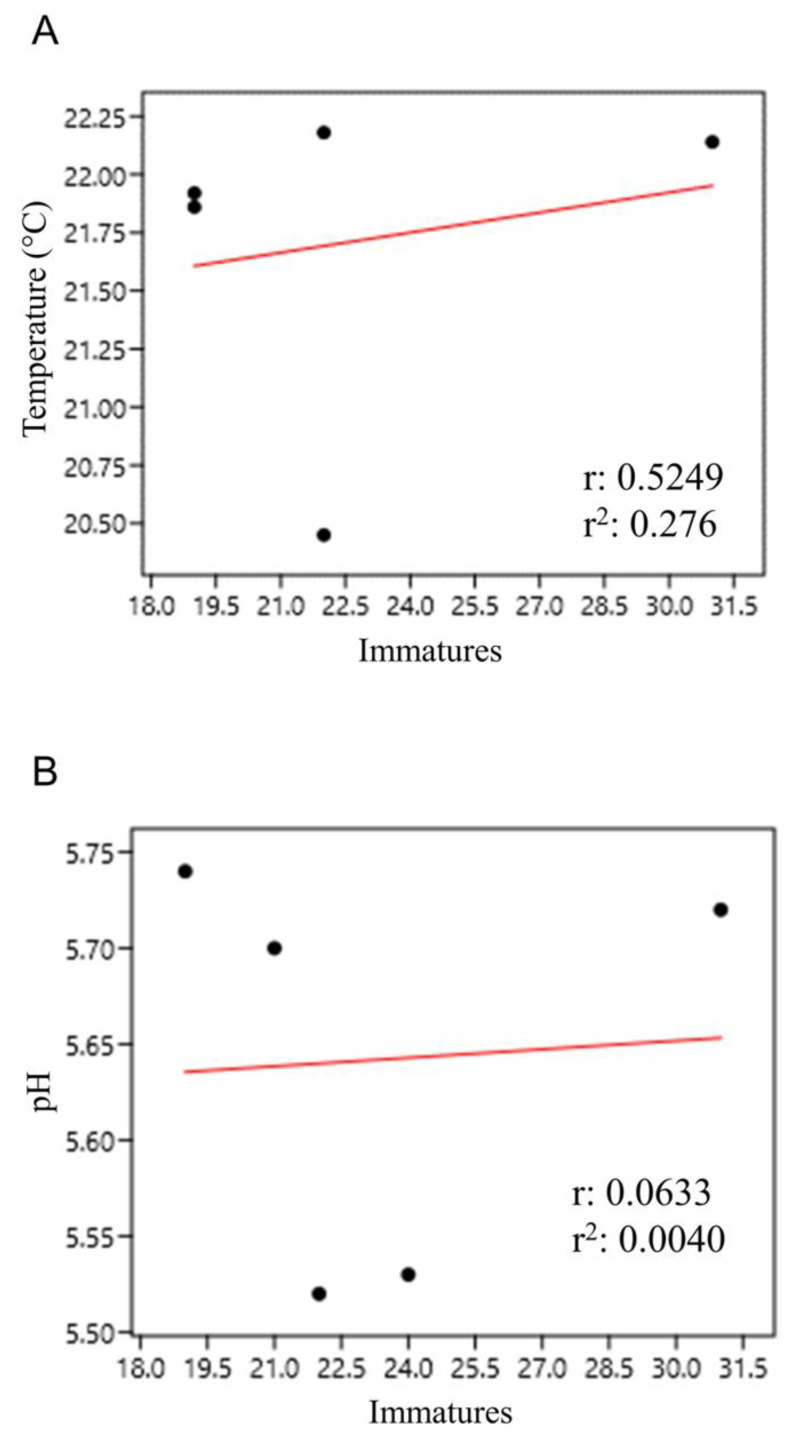
Correlation of immature abundance in bamboo internodes with temperature (**A**) and pH (**B**) at the Tijuca National Park, city of Rio de Janeiro, Brazil, from March 2022 to March 2023.

In general, immature abundance in the internodes of bamboo specimens had a weak and positive correlation with temperature (r = 0.525) and pH (r = 0.063) (Figure 10).

**Figure 11:**
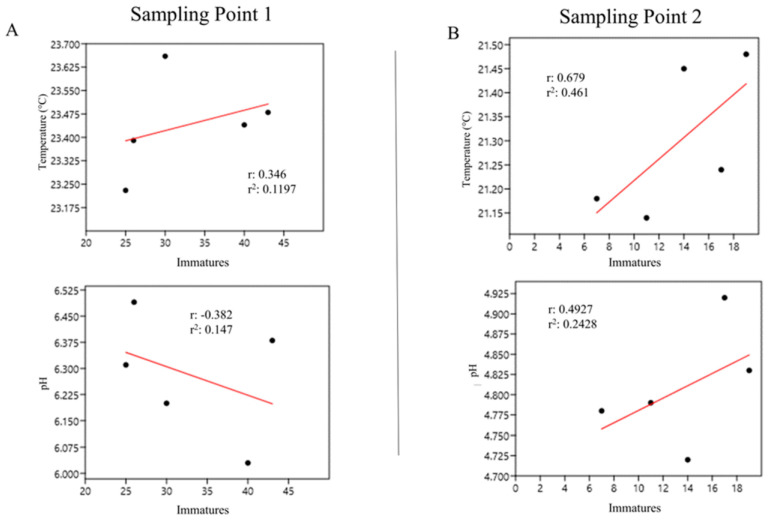
Abundance of immatures in the internodes of bamboo plants correlated with temperature and pH at sampling points 1 (**A**) and 2 (**B**) at the Tijuca National Park, city of Rio de Janeiro, Brazil, from March 2022 to March 2023.

At the internodes of bamboos from sampling point 1 (*G. tagoara*), there was a weak and positive correlation between immature abundance and temperature (r = 0.346) and a weak and negative correlation with pH (r = −0.38) (Figure 11A). It should be noted that in the internodes of bamboo at sampling point 2 (*B. vulgaris*), we found a strong and positive correlation between immature abundance and temperature (r = 0.679) and a weak and positive correlation with pH (r = 0.492) (Figure 11B).

**Figure 12:**
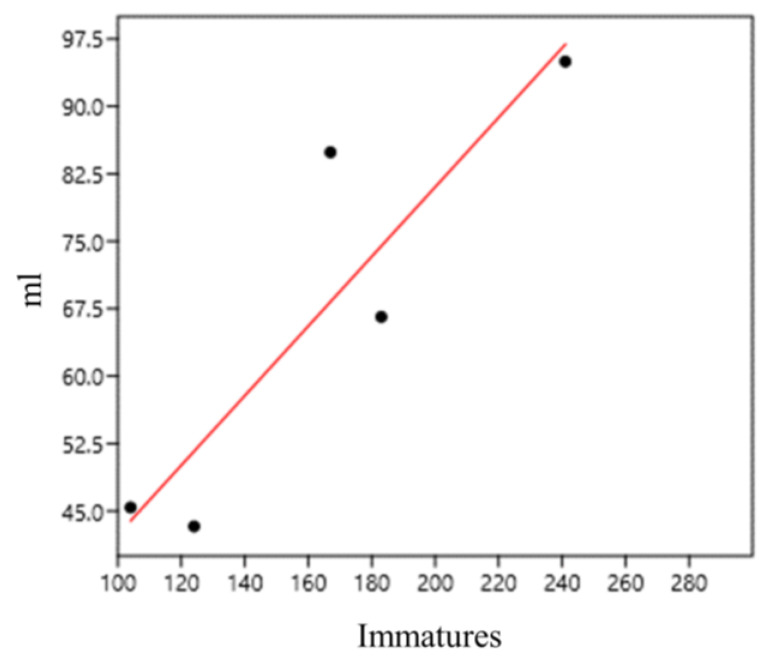
Correlation of immature abundance and water volume (mL) in *Guadua tagoara* bamboo plants (sampling point 1) at the Tijuca National Park, city of Rio de Janeiro, Brazil, from March 2022 to March 2023.

With regard to the water volume of the bamboo specimens, we found a positive and strong correlation between immature abundance and water volume (mL) per plant (0.90) at sampling point 1 (bamboo specimens 1–5; *G. tagoara*) (Figure 12). 

**Figure 13:**
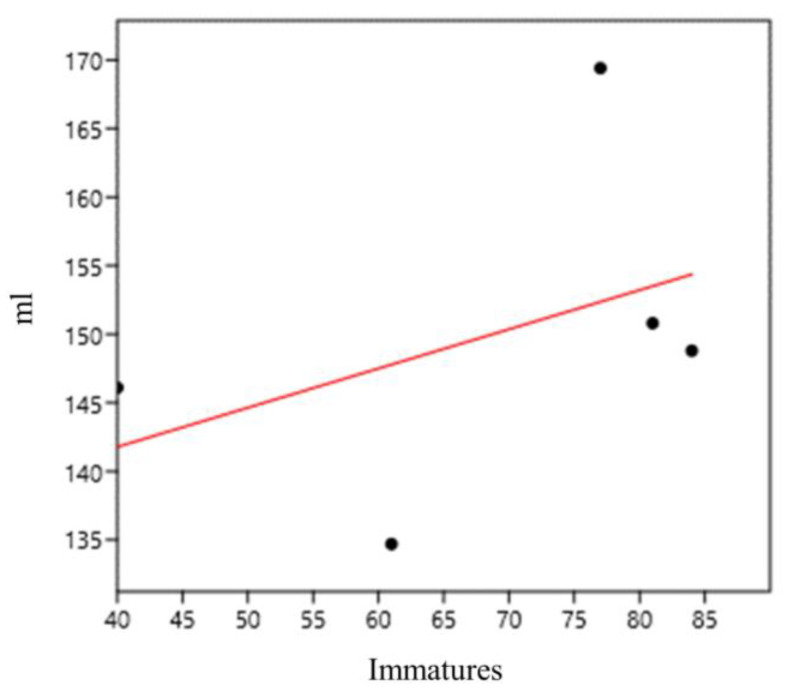
Correlation between immature abundance and water volume (mL) in *Bambusa vulgaris* bamboo plants (sampling point 2) at the Tijuca National Park, city of Rio de Janeiro, Brazil, from March 2022 to March 2023.

At sampling point 2 (bamboo specimens 6–10; *B. vulgaris*), we found a medium and positive correlation between immature abundance and water volume (mL) (0.41) (Figure 13).

**Figure 14:**
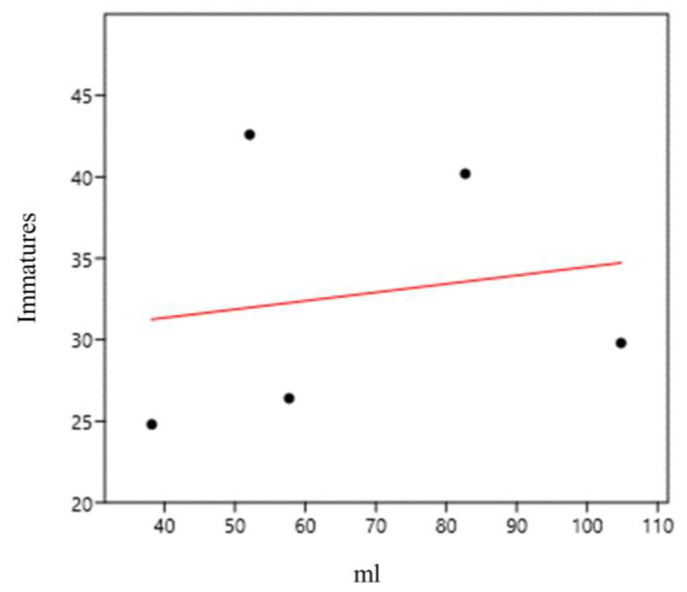
Abundance of immatures correlated with water volume (mL) in *G. tagoara* internodes (sampling point 1) at the Tijuca National Park, city of Rio de Janeiro, Brazil, from March 2022 to March 2023.

When analyzing the relationship between water volume per internode and the number of immature culicids, we noticed that at sampling point 1 (bamboo specimens 1–5; *G. tagoara*), there was a positive but weak correlation between immature abundance and water volume (mL) per internode (0.17) (Figure 14).

**Figure 15:**
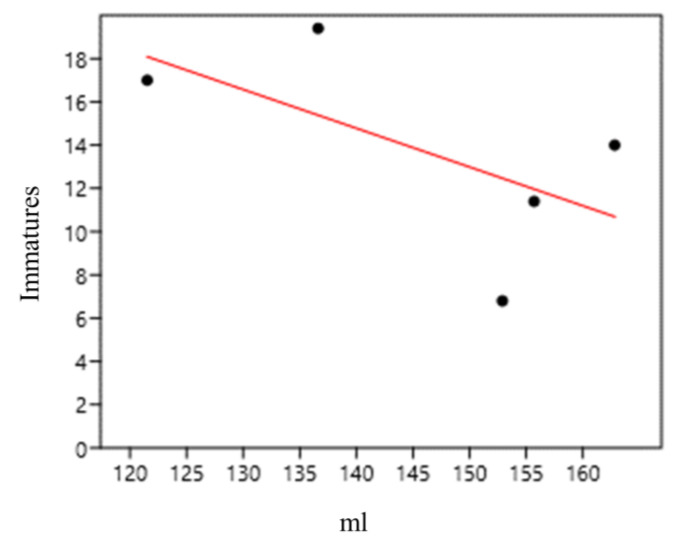
Correlation between immature abundance and water volume (mL) in *Bambusa vulgaris* internodes (sampling point 2) at the Tijuca National Park, city of Rio de Janeiro, Brazil, from March 2022 to March 2023.

At sampling point 2 (bamboo specimens 6–10; *Bambusa vulgaris*), we found a strong and negative correlation between the abundance of immatures and water volume (mL) per internode (internodes 1–5) (0.41) (Figure 15).

## 4. Discussion

In their natural environment, mosquitoes find different larval habitats formed through water accumulation in different locations. These have different levels of substrates and may or may not suffer from human interference. When analyzing the fauna that use bamboo internodes for their development, Campos (2013) [7] noted that Culicidae make up the largest number of individuals. 

Bamboo internodes are among the most specialized larval habitats for immature development of mosquito immatures (MacDonald & Traub, 1960) [5]. In addition, the holes on the side of the internodes of wild bamboo plants enable the set of transformations undergone by the substances that constitute them. Zequi and Lopes (2001) [21] emphasize that bamboo species such as *Bambusa vulgar* var. *vulgar* Schrad (usually referred to in Brazil as green bamboo), *Bambusa vulgaris* var. *vittata* Schrad (Brazilian bamboo), and *Dendrocalamus giganteus* Wallich (giant bamboo) act as important larval habitats for the maintenance of immature mosquitoes.

Lozovei (1998) [8] evaluated the mosquito species that use bamboo internodes to breed. The author found two patterns of holes produced by wildlife, circular and square/rectangular, attributing moths of the family Noctuidae (Lepidoptera) as the cause of the holes. In this experiment, Lozovei (1998) [8] reproduced similar larval habitats artificially and analyzed them simultaneously, producing transverse openings and introducing water into the reservoir, finding 17 species of dendricolous mosquitoes. 

Bastos et al. (2021) [6] studied the composition of Culicidae that breed in *Bambusa* sp. internodes, finding a richness of 17 species in a remnant of the Atlantic Forest in the state of Rio de Janeiro. This result is similar to the number of species found in the present study, in which the same pattern of circular holes in the internodes of *B. vulgaris* was used, and 16 species of mosquitoes were found during the sampling period. 

Marques and Forattini (2008) [22] point out that species abundance is related to the control one species exerts over others to become dominant. Although *Cx. neglectus*, *Sa. identicus*, *Wy. oblita*, and *Wy. lutzi* were found in all internodes, the most abundant species was *Cx. neglectus*, making it dominant. Alencar et al. (2021) [6] came to similar conclusions in their study, with *Cx. neglectus* representing 43% of the specimens collected, which is almost 10% higher than in our study. 

*Guadua tagoara* (sampling point 1) showed a higher index of diversity and equability of Culicidae compared to *B. vulgaris* (sampling point 2). In both points, species whose larvae are predatory were found, including *Sa. identicus*, *Sa. melanonymphe* (Dyar, 1924), *Tx. (Lynchiella) bambusicola* (Lutz & Neiva, 1913), *Tr. compressum* (Lutz, 1905), and *Tr. pallidiventer* (Lutz, 1905). We believe that the greater diversity of mosquito species in *G. tagoara* was due to greater exposure to them in comparison to *B. vulgaris*, which is in line with Lozovei (1998) [8], Silva et al. (2004) [23], Bastos et al. (2021) [6], and Müller et al. (2022) [10]. Therefore, the difference in diversity in the two bamboo species analyzed can be explained by the influence exerted by local environmental factors, including seasonality, competition, predation, environmental stability, and productivity. The larvae of *Toxorhynchites* species are considered to be the largest predators in internodes. A low number of *Toxorhynchites* specimens was detected in the present study. Lozovei (1998) [8] also found a low number of *Toxorhynchites* specimens on bamboo internodes in the Serra do Mar Atlantic Forest in Paraná State. Campos (2013, 2016) [7,24], evaluating the diversity of mosquitoes that use bamboo plants as larval habitats in northeastern Argentina, detected only one species of immature *Toxorhynchites* per internode, which could have occurred due to the ecological relationship of intraspecific cannibalism.

In the present study, the highest percentage of individuals who reached adulthood were males (53%). However, a monthly breakdown shows that during some months, the percentage of females exceeded that of males, which was unexpected [25,26]. Dias et al. (2023) [27] argue that locations and seasons can generate variations in proportions. They found a greater number of females in their study. Chaves et al. (2011) [26] also indicate other factors that may influence variations, such as nutrient supply and larval density.

Abiotic factors can significantly influence the development and presence of certain mosquito species. In the present study, the factors recorded were temperature and pH. Lozovei (2001) [2] mentioned that the water in the internodes can be acidic or neutral, as found in the current study. However, Campos (2013) [7] demonstrated that the water may also be strongly alkaline. Mosquitoes were found in all internodes, with pH variation in the sampling points; in sampling point 1, the pH range found was neutral, while in sampling point 2, it was an acidic range. Sampling point 1, with neutral pH, contained the largest number of individuals, which differs from the findings of Campos (2013) [7]. In that study, it was found that the largest number of species occurred with an acidic pH. Thus, such results linked to the species found may show that some will be present in both pH ranges or a single range and that it is important to link this factor to others, such as the temperature, the species of bamboo used, and the species of these mosquitoes.

Temperature is an abiotic factor that can influence larval viability, development time, and other factors associated with the life cycle of mosquitoes. The temperature was relatively similar in both locations. However, we detected higher maximum temperatures at sampling point 1 (13.4 °C and 31.8 °C) than at sampling point 2 (13.2 °C and 28.1 °C), a factor that may contribute to the increase in the mosquito population. On the other hand, there were other particularities in both locations. Sampling point 2 has a greater vegetation cover, meaning it is less exposed to the sun. The bamboo species may also influence the presence of mosquitoes. This could be seen because different species were found in different locations, and *B. vulgaris* was the only species used as a larval habitat [6].

Consideration should be given to the strategies these insects use to guarantee their survival and persistence. *Culex (Carrollia) iridescens* (Lutz, 1905) and *Cx. (Microculex) neglectus* (Lutz, 1904) were found every month, including during periods considered unfavorable due to low rainfall, such as September and December 2022.

A larger volume of water is expected to be found in the internodes during the rainy season. This would result in increased nutrient transport due to the higher water flow through the hole, making the internodes a favorable environment for the development of immatures. Our results showed that water volume influenced the number of individuals at both sampling points. This is supported by the results of the study conducted by Campos (2013) [7].

## 5. Conclusions

The Tijuca Forest National Park is a tourist spot, and it receives a large number of visitors. Humans can therefore become an accessible food source for mosquitoes in this area. Thus, considering that we found in the present study the presence of the *Hg. leucocelaenus* vector of the Yellow Fever virus in Brazil, special attention is required for the emergence of febrile illnesses among visitors to the area of the environmental conservation unit, in the surrounding communities, or even in the local population.

## Figures and Tables

**Table 1:** Diversity indices of immature mosquitoes in bamboo plants at the Tijuca National Park, city of Rio de Janeiro, Brazil, from March 2022 to March 2023.

	Sampling Point 1					Sampling Point 2				
Bamboo	1	2	3	4	5	6	7	8	9	10
Species (S)	10	8	9	9	10	7	9	7	6	8
Dominance (D)	0.23	0.36	0.28	0.31	0.24	0.25	0.24	0.31	0.27	0.42
Shannon diversity (H)	1.71	1.37	1.51	1.40	1.65	1.60	1.74	1.42	1.47	1.25
Equitability (J)	0.74	0.66	0.69	0.64	0.72	0.82	0.79	0.73	0.82	0.60

**Table 2:** Diversity indices of immature mosquitoes found in bamboo internodes at sampling point 1 (*G. tagoara*) at the Tijuca National Park, city of Rio de Janeiro, Brazil, from March 2022 to March 2023.

Internode	1	2	3	4	5
Species (S)	7	11	7	11	7
Dominance (D)	0.20	0.23	0.23	0.20	0.24
Shannon diversity (H)	1.72	1.70	1.64	1.79	1.56
Equitability (J)	0.88	0.71	0.84	0.75	0.80

**Table 3:** Diversity indices of immature mosquitoes found in bamboo internodes at sampling point 2 (*B. vulgaris*) at the Tijuca National Park, city of Rio de Janeiro, Brazil, from March 2022 to March 2023.

Internode	1	2	3	4	5
Species (S)	9	10	9	7	4
Dominance (D)	0.21	0.23	0.36	0.33	0.50
Shannon diversity (H)	1.73	1.76	1.41	1.39	0.96
Equitability (J)	0.79	0.77	0.64	0.71	0.70

**Table 4:** Diversity indices of immature mosquitoes at the Tijuca National Park, city of Rio de Janeiro, Brazil, from March 2022 to March 2023.

Month	Species (S)	Dominance (D)	Shannon Diversity (H)	Equitability (J)
March 2022	5	0.7	0.6	0.4
April 2022	10	0.4	1.5	0.7
May 2022	8	0.2	1.7	0.8
June 2022	7	0.2	1.7	0.9
July 2022	11	0.3	1.7	0.7
August 2022	8	0.2	1.6	0.8
September 2022	5	0.5	1.0	0.6
October 2022	6	0.3	1.4	0.8
November 2022	6	0.5	1.2	0.6
December 2022	7	0.3	1.4	0.7
January 2023	8	0.3	1.3	0.6
February 2023	7	0.4	1.3	0.7
March 2023	3	0.6	0.7	0.6

## Data Availability

Dataset available on request from the authors.
